# Anti-complement drugs for the treatment of geographic atrophy and the release of silicone oil

**DOI:** 10.1186/s40942-023-00523-3

**Published:** 2024-01-05

**Authors:** Gustavo Barreto Melo, Geoffrey Guy Emerson

**Affiliations:** 1 Department of Ophthalmology, Federal University of São Paulo, Rua Botucatu, 820, São Paulo, Brazil; 2 Department of Ophthalmology, University of Minnesota, Minneapolis, USA

**Keywords:** Avacincaptad pegol, Pegcetacoplan, Geographic atrophy, Silicone oil, Syringe

## Abstract

Intravitreal injections are a common procedure in ophthalmology, often using syringes coated with silicone to aid piston movement and needles coated with silicone oil to facilitate penetration of the sclera. Pegcetacoplan and avacincaptad pegol, recently approved for clinical use by the US Food and Drug Administration, have higher viscosity and seem more susceptible to entrap air bubbles compared to anti-VEGF drugs.

It is plausible that both anti-complement drugs could be associated with a higher likelihood of introducing silicone oil in the vitreous because of higher viscosity, with potentially higher friction at the inner surface of syringe barrel, in the vicinity of silicone oil. In addition to this, undesirable agitation might be inadvertently promoted by some retina specialists to remove air bubbles from the drug solution.

In conclusion, recent reports of silicone oil droplets in the vitreous of patients receiving pegcetacoplan injection might be related to both its viscosity and to agitation of the syringe to remove air bubbles. Since avacincaptad pegol also is viscous, though with different pH, syringe and filter needle, we might expect similar reports for this agent soon. We also recommend further studies be carried not only to clarify the current matter but also the potential association between the combination of agitation, silicone oil and inflammation or any immune response.

## Background

Recent publications have reported silicone oil in the vitreous of patients treated with pegcetacoplan for geographic atrophy [1–3].

To better understand this subject, one should bear in mind that most syringes are siliconized to promote a better gliding of the piston, and needles are also coated with silicone oil to better penetrate the sclera [4, 5]. Additionally, it is important to know that both pegcetacoplan and avacincaptad pegol, currently approved for commercial use by the US Food and Drug Administration, are more viscous than all other anti-vascular endothelial growth factor (anit-VEGF) drugs, and removing air bubbles entrapped in the fluid might be challenging.

## Main text

Emerson (2017) pioneered the studies addressing syringes and silicone oil for intravitreal injections [6]. He found that syringes with a dead space are less likely to release silicone oil than insulin syringes, which usually have a staked-in needle and no dead space. A dead space tends to trap silicone droplets in the syringe for two reasons. First, since silicone oil is less dense than water, when the tip of the syringe-needle setup is facing down, the oil will move upward closer to the plunger, and will mostly be trapped in the dead space. Second, because the aqueous is less viscous than silicone oil, it has an advantage over the silicone droplets in passing through the narrow cross section of the needle (while the viscous oil droplets stay behind in the dead space).

How do we explain the recent findings that silicone oil droplets are observed more often than expected in eyes after pegcetacoplan even after a single injection? Multiple reasons could be speculated, including more frequent agitation of the syringe to remove air, use of syringes with a small or absent dead space, specific physical (viscosity) and chemical properties (pH, solvent). However, two of them seem to stand out: drug viscosity and agitation of the syringe to remove air bubbles. Another potential explanation that cannot be easily ruled out is the role played by drug pH, solvent composition. Interestingly, we compared aflibercept, bevacizumab, buffer solution (as that from bevacizumab), and ziv-aflibercept [5]. We found that agitated syringes with ziv-aflibercept were statistically associated with less silicone oil release than those with buffer solution, with aflibercept and bevacizumab in the mid-range but without statistical significance.

Since both pegcetacoplan and avacincaptad pegol are more viscous than the anti-VEGF agents, it is plausible that its movement along the inner surface of the syringe barrel, where the silicone oil layer lays, might displace it more easily. Bijon et al. (2023) hypothesized that the forward-and-back movements of the plunger to remove air bubbles could potentiate the friction force over the silicone oil layer [1]. Considering the drug viscosity, it is a reasonable possibility. Against this argument, on the other hand, we previously assessed these priming movements with water and did not find any difference in the release of silicone oil [7]. Considering that water is less viscous than both anti-complement drugs, further studies are required to answer this question.

Another possibility behind the high prevalence of silicone oil droplets in the vitreous of patients receiving intravitreal injection of pegcetacoplan is agitation by flicking/tapping to remove air bubbles from the drug. Kim et al. (2020) showed that agitation of siliconized syringes leads to an increase in the release of silicone oil [8]. They also found that both the use of a silicone-free syringe and of a filter needle decreases that amount. Prior publications by our and other groups have supported the role played by agitation in the release of these silicone particles [7, 9–12]. Even ranibizumab and aflibercept prefilled syringes were found to increase the release of silicone particles after agitation [12]. We have also shown a potential association between agitation of siliconized syringes and the development of inflammation after intravitreal injection of anti-VEGF agents [13–15]. This hypothesis is also supported by laboratory studies with non-ophthalmic-use drugs in animal and cell culture models [16–18]. Although no link has been found yet between cases of occlusive retinal vasculitis and agitation of a siliconized syringe, this possibility should not be underestimated, as suggested recently [19]. Furthermore, if this were ultimately confirmed to be true, there would be a broader application of this knowledge to the whole healthcare practice [20].

## Conclusions

In conclusion, we believe this potentially higher prevalence of silicone oil droplets in the vitreous of patients receiving pegcetacoplan injection might be related to both its viscosity and to agitation of the syringe to remove air bubbles. Since avacincaptad pegol has similar features, though with different pH, syringe and filter needle, we might see similar reports of silicone droplets after its use soon. In order to minimize this complication, lubricant-free syringes are recommended, and do not flick the syringe, be it an anti-complement, antibiotic, steroid or anti-VEGF agent. We also recommend further studies to clarify the relationship between the anti-complements drugs and silicone oil as well as the potential association between the agitation, silicone oil and inflammation/immune response.

## Data Availability

Not applicable.

## Declarations

### Authors’ information

GBM is editor-in-chief of International Journal of Retina and Vitreous. This article was independently handled by a member of the Editorial Board.

## Abbreviations

Anti-VEGF: Anti-vascular endothelial growth factor
